# Factors Determining Colorectal Cancer: The Role of the Intestinal Microbiota

**DOI:** 10.3389/fonc.2015.00220

**Published:** 2015-10-12

**Authors:** Esther Nistal, Nereida Fernández-Fernández, Santiago Vivas, José Luis Olcoz

**Affiliations:** ^1^Instituto de Biomedicina (IBIOMED), Universidad de León, León, Spain; ^2^Gastroenterología, Hospital Universitario de León, León, Spain

**Keywords:** gut microbiota, colorectal cancer, bacterial metabolites, inflammation, diet

## Abstract

The gastrointestinal tract, in particular the colon, holds a complex community of microorganisms, which are essential for maintaining homeostasis. However, in recent years, many studies have implicated microbiota in the development of colorectal cancer (CRC), with this disease considered a major cause of death in the western world. The mechanisms underlying bacterial contribution in its development are complex and are not yet fully understood. However, there is increasing evidence showing a connection between intestinal microbiota and CRC. Intestinal microorganisms cause the onset and progression of CRC using different mechanisms, such as the induction of a chronic inflammation state, the biosynthesis of genotoxins that interfere with cell cycle regulation, the production of toxic metabolites, or heterocyclic amine activation of pro-diet carcinogenic compounds. Despite these advances, additional studies in humans and animal models will further decipher the relationship between microbiota and CRC, and aid in developing alternate therapies based on microbiota manipulation.

## Colorectal Cancer

According to the World Health Organization, cancer is a global health problem. In 2011, it was the leading cause of mortality positioned above stroke and coronary pathology. Approximately half of these deaths were due to lung, stomach, colon, and breast cancer.

Colorectal cancer (CRC) ranks third in incidence in men and second in women (1.4 million in 2012). It stands at third place in mortality causing up to 690,000 deaths per year (1). Approximately 75% of deaths from CRC occur in people over 65 years, with mortality higher in males. Analyzing age and sex together reveals that mortality in males is comparable to that of females who are 4–8 years older (2, 3). In Europe, CRC mortality is showing a downward trend in countries such as Austria, France, Ireland, Sweden, and Norway, unlike Eastern and Mediterranean countries such as Spain, Italy, and Greece (4). There are big differences in the incidence of CRC across countries, which are mainly attributed to diet.

Until recently, CRC was almost exclusively a public health issue in industrialized countries, but has now also become a problem in emerging countries due to economic growth (5). The adoption of a westernized lifestyle, increased consumption of red meat, high-calorie diets, and rising life expectancy in these countries have contributed to the increased incidence of CRC (6). Moreover, these countries have higher mortality due to a lack of healthcare resources (6).

About 75% of CRC cases are not hereditary and occur spontaneously, while the remaining 25% of affected individuals have a family history, which shows the combined contribution of genetics and environmental factors. However, only between 5 and 6% are due to main genetic alterations with a high penetrance (7). These facts justify the significant impact of environmental factors and lifestyle in the development of CRC. Numerous risk factors associated with CCR are analyzed: age, sex and race, diet habits, consumption of red meat, obesity, and toxic substances such as tobacco and alcohol (8, 9).

### Determinant Factors of CRC

**Figure 1:**
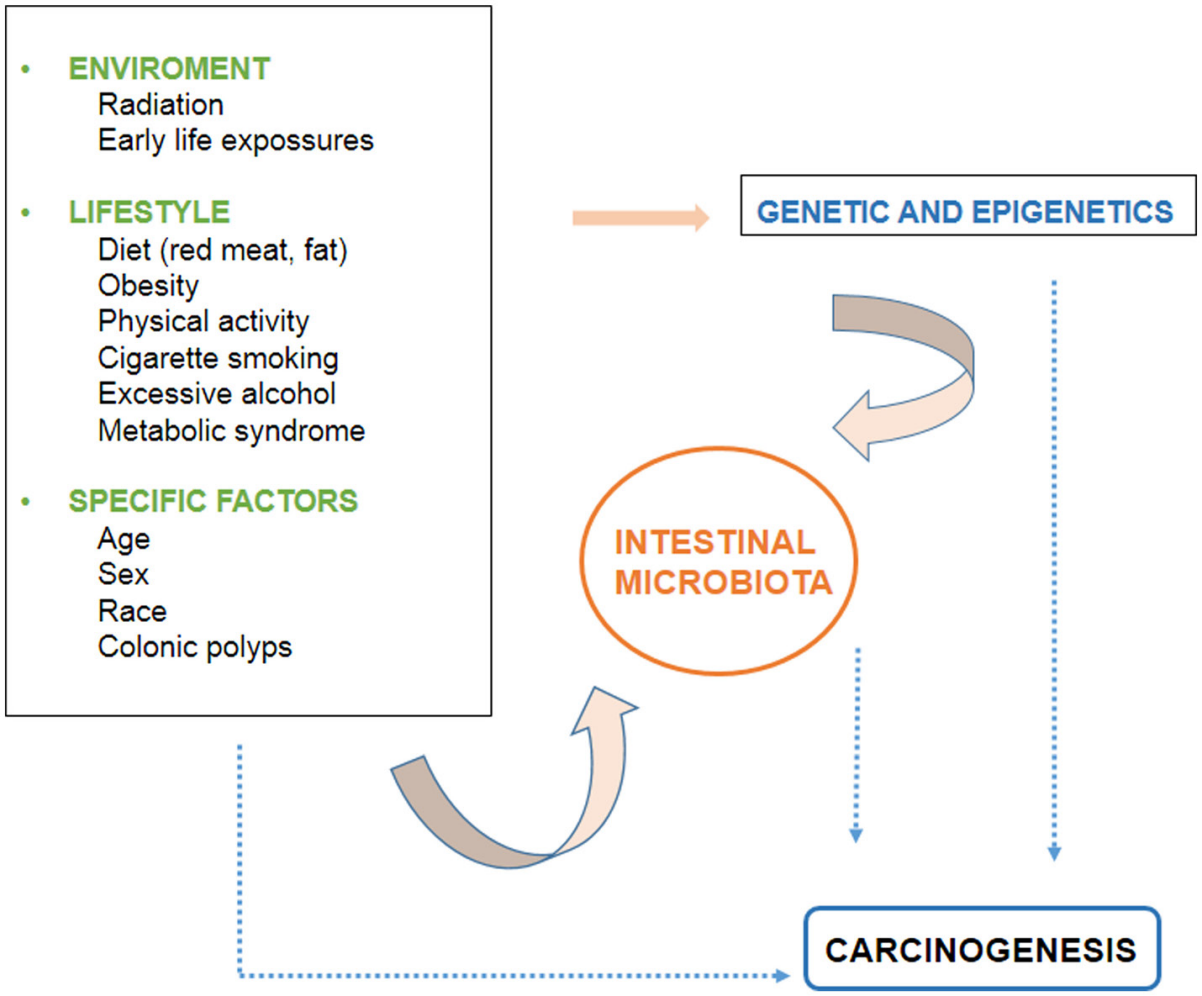
**Factors involved in the development of colorectal cancer**.

There are several environmental and individual-specific factors associated to CRC development. The evidence related to these factors is discussed below and the interactions between them are outlined in Figure 1.

The effect of diet is so important that in 1981 it was predicted that 90% of gastrointestinal cancers are due to differences in diet (10). A study published in the 90s focused on this aspect, and the rarity of CRC in the African race was related to the absence of consumption of aggressive factors, such as animal flesh and fat within this ethnic group (11). This is evident, for example, in immigration studies showing high rates of CRC in people who migrate from low-incidence countries to high-risk areas (12, 13). One example of this is that CRC incidence in Japanese people emigrating to the United States of America (USA) is similar to that of Caucasians living in the USA, and is three to four times than that of Japanese people living in Japan. Similar results were found for native Africans and Afro-American people; although CRC incidence in the first group is much lower than that in the latter, mainly due to different environmental factors, especially diet habits (14). The important role of environmental factors is also evidenced in the age structure of CRC in developing countries. These countries have a high CRC incidence in young age ranges with lower levels in the oldest group. This suggests that recent lifestyle changes have increased the risk of young people to develop CRC. Such changes are related to diet habits, particularly increased fat and meat consumption (15, 16). There is evidence that red meat and animal fat are associated to an increased CRC risk (17).

A sedentary lifestyle, a Western diet rich in meat and fat and low in fiber, increases the risk of CRC. The absence of physical activity, increased caloric intake, and changes in eating patterns to a more westernized one were omnipresent settled in Eastern Asia, Europe, and North Africa, implying a parallel rise in obesity and in CRC incidence in these emerging countries (5, 18). Multiple studies support the idea of an increased risk of adenomas and CRC in patients with obesity and metabolic syndrome, which are associated to economic growth and globalization (19, 20). It seems that insulin-like growth factor, IGF-1, which is higher in obese people with insulin resistance, promotes cell growth and inhibits apoptosis pathways leading to carcinogenesis (21).

Toxic habits such as tobacco and alcohol consumption are also associated to an increase in the risk for CRC because they cause chronic inflammation (22). Tobacco is particularly important since it also acts at other levels causing increased oxidative stress, genetic, and epigenetic alterations (19).

Genetic mutations, epigenetic changes and alterations in immunogenic signaling pathways associated and modified by the environment, are major contributors to CRC (23). The molecular mechanisms of interaction between the environment, genetics, and carcinogenesis are very complex and are not yet fully understood. The genetic alterations that lead to the development of CRC have been extensively studied in recent decades. Current knowledge has mainly been obtained from studies on Western populations. Although the main signaling pathways are similar in developing countries, the spectrum of mutations may differ due to different environmental or genetic factors (14).

Epigenetic regulation plays a fundamental role in CRC initiation and progression (23). Epigenetic alterations that involve DNA methylation, histone changes, or modification of antisense RNA can alter gene expression alone, or in combination with inherited or somatic mutations. For instance, alterations in methylation of certain CpG dinucleotides can inactivate the development of tumor suppressor genes (24). These epigenetic alterations can be strongly affected by diet habits or chronic alcohol consumption. Some food components cause changes in gene methylation (19).

There is a clear interaction between lifestyle, environmental factors, genetic, and epigenetic alterations. Certain lifestyles are associated with a CRC molecular subtype. Since obesity was associated with an increased risk of CRC, there have been many studies searching for the molecular pathways to prove this relationship. A recent meta-analysis demonstrated the inverse relationship between adiponectin levels and the risk of CRC (25). By contrast, leptin, which is present in high levels in obese people, is directly related to CRC risk. Another example is that the risk of developing tumors in subjects with the K-RAS mutation and p53 was lower in patients with increased physical activity. Even tobacco consumption was associated with an increased risk of microsatellite instability, BRAF mutations, and CIMP-positive tumors, which shows the relationship between tobacco and epigenetic alterations (26). Consumption of red meat and a high glycemic load, as in Western diet, was associated with the CRC-p53 mutation (27).

Although there is evidence that diet influences the development of CRC at the molecular level, it is not completely clear since the mechanism is highly complex, and requires the interaction of a large number of elements, as mentioned above. The interaction of dietetic factors in the gastrointestinal lumen and colonic epithelium is regulated by the genetic susceptibility of the individual. In addition, the microbiota is recently emerging as an increasingly important factor. It is believed that the intestinal component whose products determine the health of the colon and the individual, and interact with environmental factors and the genetics of the person (Figure 1).

## Colonic Microbiota

The emergence of molecular sequencing techniques is a major breakthrough for the identification of intestinal microorganisms that could not have been isolated and characterized by traditional culture methods (28–31). These molecular techniques are based on detecting differences in nucleotide sequences of various microbial genes. To do this, DNA is extracted from the sample, followed by amplification and sequencing of genes coding for the 16S ribosomal RNA subunit. A recent development is “metagenomics,” where the genetic materials of samples of a particular ecological niche are recovered and directly analyzed, thus obviating the need for isolating and culturing different member organisms (32). Furthermore, this technique not only has the advantage of phylogenetic characterization of a microbial community, but also provides information on the biological functions that are present in that community.

### Colon Microbiota Composition

The gastrointestinal tract is the natural habitat of large, dynamic, and diverse populations of microorganisms, mainly bacteria that have adapted to life on mucosal surfaces or in the intestinal lumen (33, 34). The presence of each organism depends on the morphological and physiological characteristics of respective regions of the digestive system (35). This microbiota grows in number and complexity as we move through the gastrointestinal tract. The large intestine is the main colonization niche in the human body. It is estimated that the colon houses about 10^14^ microbial cells, most of them bacteria. *Bacteroidetes* and *Firmicutes* are the dominant phyla in the large intestine, followed by *Actinobacteria* and *Verrucomicrobia*. The phylum *Proteobacteria* is also present, but to a lesser extent (36). Factors that facilitate bacterial growth in the colon are the increase in pH, reaching neutrality, and the reduction of both bile salt concentration and traces of pancreatic secretion. Furthermore, transit time in the colon is slow, providing microorganisms with the opportunity to proliferate, and ferment available substrates derived from diet or endogenous secretions (37, 38). The colon has a reductive environment devoid of oxygen. Thus, most microbial populations are strictly anaerobic. Within this microbiota, the *Bacteroides* genus is one of the most abundant (39). Gram-positive non-spore-forming microorganisms such as *Eubacterium*, *Bifidobacterium*, *Peptostreptococcus*, and *Ruminococcus* are also dominant (39). Spore-forming Gram-positive bacilli are mainly represented by the genus *Clostridium*. To a lesser extent, facultative anaerobes or aerotolerant ones such as enterobacteria, enterococci, lactobacilli, and streptococci, which are essential for microbial homeostasis, appear in the large intestine. Differences were observed in the composition between the microbiota that is present in the intestinal lumen, and the one associated with the mucosa, but their biological significance is still unclear (40, 41).

### Microbiota Functional Capacity

The intestinal microbiota acts as a “metabolic organ” that interacts with the human host and performs many essential functions required for the maintenance of human health (42). These metabolic functions allow guests to use available energy sources such as carbohydrates and proteins. In return, the microbiota produce vitamins, synthesize amino acids, influence the absorption of ions, participate in the conversion of dietary polyphenolic compounds, and are involved in the biotransformation of bile acids (43–45). Associated with these metabolic functions, the intestinal microbiota is essential for the development of the immune system, helping it maintain the function of the intestinal barrier and assisting in an adequate immune response to pathogens (46–48). This symbiotic relationship with the host is key for maintaining the balance of the gut microbiota in the intestine. A change in that balance (dysbiosis) under abnormal conditions may cause adverse consequences for the host. This intestinal dysbiosis may be associated with the overgrowth of opportunistic pathogens that are normally inhibited by commensal bacteria (49). It has been described in inflammatory digestive diseases (50–52), obesity (53–55), colorectal adenomas, and cancer (56–60).

## Microbiota and Colon Cancer

Colon cancer is the result of a multifactorial process whose progression is associated with the gradual accumulation of genetic and epigenetic mutations. In more than 90% of these cases, it is sporadic and develops gradually with the appearance of adenomatous polyps in the epithelium, leading to an invasive carcinoma. It is a slow process, which can last up to 10 years, depending on mutation frequency (61). Besides genetic factors, environmental factors associated with the onset of colon cancer, such as chronic inflammation or diet habits, are also implicated (62, 63). Nevertheless, in recent years studies have implicated the intestinal microbiota in the development of this disease (64–66).

### Intestinal Dysbiosis and CRC

Initially, colon cancer was associated with certain pathogenic species such as *Streptococcus gallolyticus*, *Helicobacter pylori*, or *Escherichia coli* (67, 68). However, a number of studies over the past decade maintain that there are multiple bacterial species that contribute to CRC. Alterations in the composition, distribution, and metabolism of the microbiota in the colon may cause changes in homeostasis resulting in the onset of inflammation, dysplasia, and cancer (69). The main gut bacteria implicated in dysbiosis, found in several studies, are listed in the Table 1 and discussed below.

**Table 1:** **Gut bacteria associated with dysbiosis in CRC**.

Microorganism	Changes in microbiota	Sampling	Reference
*Fusobacterium*	****↑****	Feces/Mucosa	(56, 60, 70)
*Enterococcaceae*	****↑****	Feces	(60, 71)
*Campylobacter*	****↑****	Feces	(60)
*Erysipelotrichaceae*	****↑****	Feces	(60, 72)
*Collinsella*	****↑****	Feces	(60)
*Peptostreptococcus*	****↑****	Feces/Mucosa	(56, 60)
*Anaerotruncus*	****↑****	Feces	(60)
*Faecalibacterium*	****↓****	Feces/Mucosa	(56, 60)
*Roseburia*	****↓****	Feces	(56, 60)
*Porphyromonas*	****↑****	Mucosa	(56)
*Mogibacterium*	****↑****	Mucosa	(56)
*Blautia*	****↓****	Mucosa	(56)
*Bifidobacterium*	****↓****	Mucosa	(56, 73, 74)

Using next generation sequencing techniques, several studies have been conducted to compare fecal samples and luminal intestinal microbiota of CRC patients and healthy individuals (40, 56, 60, 66). These studies have shown greater microbial richness in patients with rectal adenomas compared to control individuals, where *Pseudomonas*, *Helicobacter*, and *Acinetobacter* genera appear, suggesting that these pathogens can disrupt the intestinal environment, for instance, by causing pH changes, described in association with *Helicobacter* (59). Increases in *Fusobacterium*, *Enterococcaceae*, *Campylobacter*, *Erysipelotrichaceae*, *Collinsella*, *Peptostreptococcus*, and *Anaerotruncus* have also been seen in fecal samples from CRC patients compared to healthy control individuals, opposite to a decrease of cluster IV and XIV *Clostridium* members, such as *Faecalibacterium prausnitzii* and *Roseburia*, both butyrate-producing bacteria. CRC patients showed an increase of *Porphyromonas*, *Fusobacterium*, *Peptostreptococcus*, and *Mogibacterium* in the intestinal mucosa, while *Faecalibacterium*, *Blautia*, and *Bifidobacterium* appeared diminished. Therefore, microbiota associated to sick patients is enriched with pro-inflammatory opportunistic pathogens such as *Fusobacterium*, *Campylobacter*, and *Enterococcaceae* (70, 71), as well as, microorganisms commonly associated with metabolic disorders, such as *Erysipelotrichaceae* (72). By contrast, strategic patterns that preserve microbial intestinal homeostasis are decreased, as in the case of butyrate-producing bacteria and bifidobacteria (73, 74). Overall, these data reflect the ecosystem of the intestinal microbiota of patients with CRC, i.e., a pro-inflammatory profile that can modify the existing mutual relationship between the microbiota and the host, eventually lead to a state of disease (65). An enrichment of microorganisms specifically localized to the tumor area was also seen by comparative analysis between the existing microbiota in cancerous mucosal tissues and surrounding tissues (56, 66, 75, 76). Burns et al. (75), in addition to showing increased microbial diversity in the tumor area, also described an enrichment of virulence-associated bacterial genes in the tumor microenvironment, supporting the hypothesis that the microbiome plays an active role in CRC development and/or progression. On the other hand, it has also been demonstrated for the first time, that bacterial biofilms are associated with CRCs. These bacterial biofilms were associated with diminished colonic epithelial cell E-cadherin and enhanced epithelial cell IL-6 and STAT3 activation, as well as increased crypt epithelial cell proliferation, in normal colon mucosa. The risk of developing CRC is higher in patients with biofilms compared to those without them (77).

However, these studies do not reflect whether intestinal dysbiosis is a cause or consequence of the disease. Nor do they provide information about the mechanisms through which the intestinal microbiota influence the development of CRC, or more precisely, the trigger that gives the microbiota a carcinogenic profile. In order to answer this question, the role that microorganisms play at the beginning of CRC was explored. Tjalsma et al. (78) proposed a dynamic model that explains how the microbiota is involved in the onset and progression of CRC, which he called the “bacterial driver-passenger.” According to this model, certain populations of bacteria (drivers) with pro-carcinogenic features are able to initiate the development of the disease, by damaging the DNA in the intestinal epithelium. Following tumorigenesis initiation, an alteration of the intestinal environment occurs, resulting in the overgrowth of opportunistic bacteria (passengers) and a decrease in pioneer strains. Passenger bacteria are always autochthonous members of the gut microbial community, are relatively poor colonizers of a healthy intestinal tract, and show a competitive advantage in the tumor microenvironment, since they are capable of promoting tumor progression. For instance, nutrients and co-factors specific to the tumor microenvironment – such as reactive oxygen species – can be selectively utilized by specific bacterial passengers. However, in contrast to drivers, which are always pro-carcinogenic, passenger bacteria can be either pro-carcinogenic or protective, depending on the microorganism (78).

### Possible Mechanisms of Action of Intestinal Microbiota in CRC Pathogenesis

**Figure 2:**
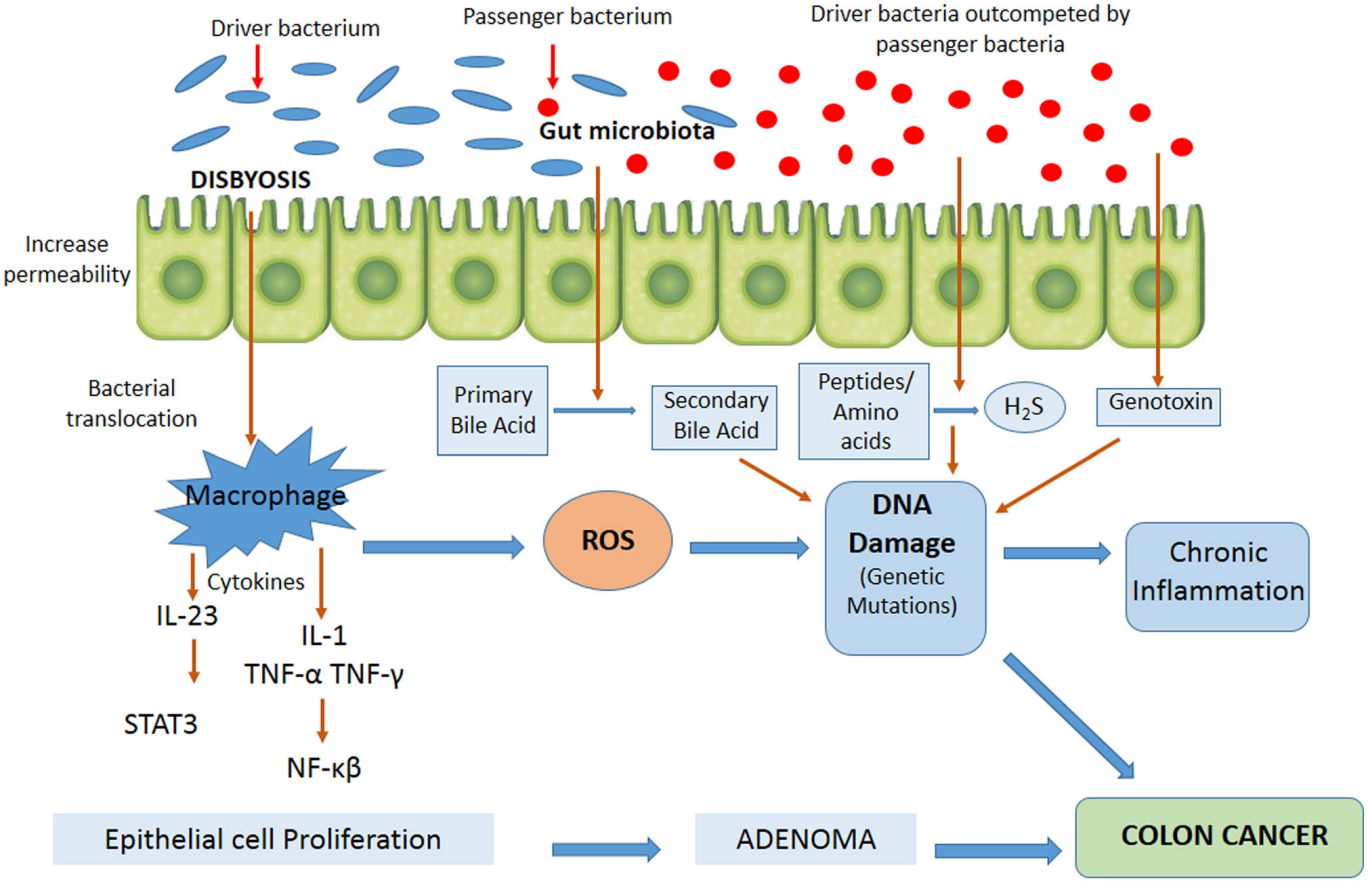
**Mechanisms possibly involved in microbial colorectal cancer promotion and the dynamic model “bacterial driver-passenger.”** Gut microorganisms may promote colorectal cancer onset and progression by different processes, such as the induction of a chronic inflammatory state, the biosynthesis of genotoxins interfering with the cell cycle regulation or directly damaging DNA, or the production of toxic metabolites. According to the model, “bacterial driver-passenger” occurs a change in the composition of the gut microbiota. At the beginning of colorectal cancer progression, driver bacteria are able to initiate the development of the disease. Following tumorigenesis initiation, an alteration of the environment occurs, resulting in the overgrowth of passenger bacteria. ROS, reactive oxygen species; STAT3, signal transducer and activator of transcription 3; H_2_S, hydrogen sulfide.

Figure 2 contains the factors involved in CRC development with possible mechanisms associated to the role of intestinal microbiota in tumor pathogenesis. In recent years, it has been observed that intestinal microorganisms can promote the onset and progression of CRC by different processes, such as the induction of a chronic inflammation state, genotoxin biosynthesis that interfere with cell cycle regulation, toxic metabolite production, or heterocyclic amine activation of pro-diet carcinogenic compounds (65). Chronic inflammation is associated with a high risk of developing cancer and does so by inducing mutations, inhibiting apoptosis or stimulating angiogenesis and cell proliferation (79, 80). An imbalance of microbiota in favor of opportunistic pathogens contributes to higher mucosa permeability, bacterial translocation, and the activation of components of both the innate and adaptive immune systems, resulting in chronic inflammation (81). The activation of the innate immune system by commensal bacteria results in an increased release of pro-inflammatory cytokines by macrophages, dendritic cells, and “natural killer” cells, such as IL-12, IL-23, TNF-α, and INFγ, with subsequent activation of cells of the adaptive immune system, including lymphocytes, both T and B cells, and various inflammatory mediators (48). One of the major consequences of this inflammatory response to commensal bacteria is the activation of transcription factors of specific key cellular signaling pathways, such as NF-κB and STAT3, in epithelial cells (82–85), and the generation of reactive oxygen and nitrogen species, leading to oxidative stress, DNA damage, aberrant proliferation, and, finally, the development of colorectal adenomas and cancer. It has been shown that colonization of germ-free animals with *Enterococcus faecalis* and *Bacteroides vulgatus* leads to the activation of NF-κB signaling pathways in epithelial cells (86). Colonization of germ-free mice with microbiota from tumor-bearing mice significantly increased tumorigenesis in the colon, compared to animals colonized with healthy gut microbiota (87). Whereas colonization of germ-free mice with human microbiota (CRC patients and healthy individuals) suggests that the initial structure of the microbiome determines susceptibility to tumorigenesis in the colon (88). Therefore, to date, these studies suggest that the alteration of normal homeostasis between host and microbiota is crucial for inflammation and consequently a number of changes occur that lead to colon carcinogenesis. Furthermore, it has been found that colonic polyposis is associated with high densities of microorganisms accumulated inside polyps, which trigger local inflammatory response. The inflammatory responses, as well as microbial density and polyp growth, can be suppressed by IL-10, specifically derived from T and T-regs cells (89).

Intestinal microbiota has also been associated with CRC development, due to toxic or genotoxic bacterial metabolite production that may lead to mutations by binding to specific cell surface receptors and affecting intracellular signal transduction (90). These toxins affect many key eukaryotic processes, such as cell signaling, and in some cases, can directly attack the genome, damaging DNA and undertake an enzymatic attack or an indirect attack causing an inflammatory reaction resulting in the release of free radicals. It has also been seen that they can affect DNA repair mechanisms (65). For instance, *Bacteroides fragilis* enterotoxigenic strains can colonize the human mucosa in an asymptomatic way, but in some cases they release the *B. fragilis* toxin (BFT) that can cause inflammatory diarrhea. BFT is a zinc-dependent metalloprotease that quickly alters the structure and function of epithelial cells of the colon, including the breaking of the tumor suppressor protein, E-cadherin (91–93). E-cadherin is a calcium dependent cell–cell adhesion molecule with pivotal roles in epithelial cell behavior, tissue formation, and cancer suppression (94). The loss of this protein increases the permeability of polarized epithelial cells in the colon, this being one of the first tumor development steps (95). BFT also activates the transcription factor NF-κB resulting in the release of cytokines that contribute to mucosa inflammation (96). However, although BFT is considered one of the main toxins in CRC development, recent studies have shown that toxins that are most actively transcribed in tumor tissues of CRC patients are derived from *E. coli*, *Salmonella enterica*, and *Shigella flexneri*. This suggests the strong involvement of enterobacterial toxins in tumorigenesis (97). Certain commensal *E. coli* strains are able to produce colibactin toxin directly in the colon. This toxin causes double-strand breaks in the DNA of colon cells. Cellular repair systems fail to restore these DNA lesions, which result in an accumulation of chromosomal anomalies and, thus, mutations leading to tumorigenesis (98).

The effect of diet on colorectal adenomas and cancer has been studied in detail. Diet mainly affects the composition and activity of intestinal bacteria (99). Therefore, the connection between diet and CRC can be partly explained, from intestinal microbiota activities. Fermentation of complex carbohydrates by colonic bacteria releases SCFAs, such as butyrate, which are an energy source for colonocytes (100) and are demonstrated to have a protective role preventing the development of CRC (101–103). Butyrate promotes many of the large intestine functions, such as colon motility, improving visceral blood flow, and preventing the overgrowth of pathogens (104–107). It also reduces inflammation, induces apoptosis, and inhibits tumor cell progression (104, 105, 108, 109). Several studies showed that the presence of fiber in the diet influences SCFA production (110). Therefore, a fiber-rich diet results in a significant increase in SCFAs, with a consequent reduction in intestinal pH, which favors colonic fermentation, prevents pathogen colonization, and decreases carcinogen absorption (106), thus reducing CRC risk (48).

It has been seen that gut bacteria contribute to nutrient metabolism and produce small molecules, which may contribute to the development of neoplastic cells in the large bowel, since metabolic differences are seen during the analysis of rectal mucosa biopsies from individuals with and without adenomas. Nugent et al. (58) described the existence of around 23 metabolites in adenomas, some of them with an inflammatory nature, such as PGE2, and a decrease in antioxidant metabolites, such as, 5-oxoproline and diketogulonic acid. Several microbial metabolites have been identified as potential carcinogens, particularly secondary bile acids that were detected at high levels in fecal samples from CRC patients (111).

The intestinal microbiota plays an important role in the metabolism of bile acids, mainly in the 7α-hydroxylation process, where cholic acid is converted into deoxycholic acid while chenodeoxycholic acid turns into lithocholic acid. This transformation increases the hydrophilicity of these secondary bile acids (112). It was shown that deoxycholic acid damages intestinal tract mucosa and contributes to an increase in reactive oxygen species, damaging DNA, generating genomic instability, and benefiting tumor growth – a process that could be key in the effect of bile acids on colon carcinogenesis (113). Secondary bile acids may also influence CRC by supporting of apoptosis-resistant cells or by interacting with important secondary messengers of the signaling system that are activated in CRC (114).

Some other metabolites that may also be involved in CRC development are protein fermentation-derived products (65). If an increase in protein intake occurs, there is an increase of waste in the colon, such as sulfide, nitrate, ammonium, amines, branched-chain amino acids, and H_2_S. Such waste resulting from protein digestion can stimulate the growth of sulfate-reducing bacteria, such as *Desulfovibrio* and *Desulfomonas* spp. (115). H_2_S, an end product of protein metabolism, was shown to be pro-inflammatory and genotoxic (116). In addition, CRC patients have a higher concentration of H_2_S compared to healthy subjects (117), and their colons have decreased ability to detoxify, thus promoting genotoxic effects (118, 119). Another bacterial subgroup, which includes several species of *Bacteroides* genus and some *Firmicutes*, is responsible for the fermentation of aromatic amino acids leading to potentially bioactive products, such as phenylacetic acid, phenols, indoles, and p-cresol. Some of these nitrogen products, particularly N-nitrous compounds (NOCs), have a potential role in causing cancer and exert their carcinogenic effect by alkylating DNA, leading to mutations. Consumption of NOCs in the diet is positively correlated with CRC (120), although these compounds may also be endogenously formed in the stomach or from nitrosation of amines that are derived from proteins fermented by existing microbiota in the large intestine. An increase in NOCs in the feces of individuals on a protein-rich diet has been described (121). Therefore, higher consumption of red meat or processed meat, compared to fruits and vegetables, is associated with an outgrowth of bacteria that might contribute to a more hostile gut environment, and is generally considered a risk factor in the development of colorectal adenomas and cancer (122–125). Bacteria metabolize meat proteins and produce nitrosamines, which are considered promoters of colon tumors that can be seen in animal models (126).

## Conclusions and Future Guidelines

All above studies suggest that different microbial metabolites generated from the metabolism of carbohydrates, proteins, and lipids could contribute to colorectal carcinogenesis through different mechanisms. It is necessary to undertake additional studies, in humans and animal models, to decode and understand the mechanisms underlying the interaction between diet, microbiota, and CRC. This will allow us to manipulate the microbiota to prevent or treat CRC (48). The use of probiotics is an alternative that has increased in popularity in recent years. A large number of studies conducted both *in vitro* and in animal models showed that consumption of probiotics could prevent CRC. In addition, treatment with probiotics increased the density and diversity of mucosal microbes, and altered the mucosa-associated microbiota in patients with CRC (127). It has also been seen that its use in CRC patients can influence innate immune system routes, and modulate apoptosis, reduce oxidative stress, and change intestinal bacteria composition and metabolism (69, 128). Although a number of laboratory studies exist, only a limited number of clinical trials have evaluated the potential role of probiotics in suppressing CRC. Probiotics most frequently used in clinical trials are species of the *Lactobacillus* genus. For example, it was found that *Lactobacillus johnsonii* reduced the concentration of intestinal *Enterobacteriaceae* and modulated the immune response in CRC patients (129). Other research showed that *Lactobacillus casei* reduced the growth of colorectal tumors in patients after 2–4 years of treatment. However, these trials are limited by patient numbers and their short lengths (130). Thus, it is necessary to perform additional studies to further support the clinical use of probiotics as preventative treatment for CRC.

## Conflict of Interest Statement

The authors declare that the research was conducted in the absence of any commercial or financial relationships that could be construed as a potential conflict of interest.
